# Augmented *Pla2g4c/Ptgs2/Hpgds* axis in bronchial smooth muscle tissues of experimental asthma

**DOI:** 10.1371/journal.pone.0202623

**Published:** 2018-08-30

**Authors:** Yoshihiko Chiba, Wataru Suto, Hiroyasu Sakai

**Affiliations:** 1 Department of Physiology and Molecular Sciences, School of Pharmacy, Hoshi University, Tokyo, Japan; 2 Department of Analytical Pathophysiology, School of Pharmacy, Hoshi University, Tokyo, Japan; University of Houston, UNITED STATES

## Abstract

**Rationale:**

Augmented smooth muscle contractility of the airways is one of the causes of airway hyperresponsiveness in asthmatics. However, the mechanism of the altered properties of airway smooth muscle cells is not well understood.

**Objectives:**

To identify differentially expressed genes (DEGs) related to the bronchial smooth muscle (BSM) hyper-contractility in a murine asthma model.

**Methods:**

The ovalbumin (OA)-sensitized mice were repeatedly challenged with aerosolized OA to induce asthmatic reaction. Transcriptomic profiles were generated by microarray analysis of BSM tissues from the OA-challenged and control animals, and KEGG (Kyoto Encyclopedia of Genes and Genomes) Pathway Analysis was applied.

**Measurements and main results:**

Tension study showed a BSM hyperresponsiveness to acetylcholine (ACh) in the OA-challenged mice. A total of 770 genes were differentially expressed between the OA-challenged and control animals. Pathway analysis showed a significant change in arachidonic acid (AA) metabolism pathway in BSM tissues of the OA-challenged mice. Validation of DEGs by quantitative RT-PCR showed a significant increase in PLA_2_ group 4c (*Pla2g4c*)/COX-2 (*Ptgs2*)/PGD_2_ synthase 2 (*Hpgds*) axis. PGD_2_ level in bronchoalveolar fluids of the OA-challenged mice was significantly increased. A 24-h incubation of BSM tissues with PGD_2_ caused a hyperresponsiveness to ACh in naive control mice.

**Conclusions:**

AA metabolism is shifted towards PGD_2_ production in BSM tissues of asthma. Increased PGD_2_ level in the airways might be a cause of the BSM hyperresponsiveness in asthma.

## Introduction

Enhanced airway responsiveness to non-specific stimuli, called airway hyperresponsiveness (AHR), is a characteristic feature of bronchial asthma. One of the causes of the AHR is hyper-contraction of smooth muscle cells of the airways [1–5]. Rapid relief from airway limitation in asthma attack by short-acting beta_2_-stimulant inhalation may also suggest an involvement of augmented airway smooth muscle contraction in the airway obstruction. It is thus important for development of asthma therapy to understand the disease-related changes in the contractile signaling of airway smooth muscle cells.

Smooth muscle contraction is caused by the interaction of myosin and actin filaments, and regulated by various contractile and Ca^2+^-sensitizing proteins [6, 7]. One possible explanation of the hyper-contraction of smooth muscle may be an up-regulation of these proteins associated with contraction. In addition to their contractile function, smooth muscle cells of the airways also have ability to generate/secrete various biologically active substances including interleukins, chemokines, and prostanoids [8–13]. The airway structural cells, such as epithelial cells (*e*.*g*., [14]), and the accumulated inflammatory cells, such as eosinophils (*e*.*g*., [15]), also release various mediators around the smooth muscle cells. Some of these autocrine/paracrine mediators affect transcriptional signaling in the cells of airway smooth muscle, resulting in an alteration of its function [12, 16–19]. Thus, an inclusive analysis of differentially expressed genes in airway smooth muscle tissues of asthma might provide new insight into the treatment of the AHR.

In the present study, we used a well-characterized asthma model of mice, which have AHR both *in vivo* and *in vitro* [20]. A microarray analysis was applied to identify the differentially expressed genes in BSM tissues of the AHR animals. Gene Ontology (GO) and Kyoto Encyclopedia of Genes and Genomes (KEGG) pathway analyses revealed the arachidonic acid metabolism pathway as a significantly changed pathway associated with AHR. In particular, an augmentation of phospholipase A_2_ group 4c (*Pla2g4c*)/cyclooxygenase-2 (*Ptgs2*)/prostaglandin D_2_ synthase 2 (*Hpgds*) cascade was strongly suggested. Thus, the role of prostaglandin D_2_ (PGD_2_) in the development of BSM hyperresponsiveness, one of the causes of AHR, was also investigated.

## Materials and methods

### Animals and treatments

Male BALB/c mice were purchased from the Tokyo Laboratory Animals Science Co., Ltd. (Tokyo, Japan) and housed in a pathogen-free facility. All animal experiments were approved by the Animal Care Committee of the Hoshi University (Tokyo, Japan).

Preparation of a murine model of allergic bronchial asthma, which has an *in vivo* AHR [20], was performed as described previously [18, 21]. In brief, BALB/c mice (8 weeks of age) were actively sensitized by intraperitoneal injections of 8 μg ovalbumin (OA; Seikagaku Co., Tokyo, Japan) with 2 mg Imject Alum (Pierce Biotechnology, Inc., Rockfold, IL, USA) on Day 0 and Day 5. The sensitized mice were challenged with aerosolized OA-saline solution (5 mg/mL) for 30 min on Days 12, 16 and 20. A control group of mice received the same immunization procedure but inhaled saline aerosol instead of OA challenge. The aerosol was generated with a compressor nebulizer (MiniElite^™^: Philips Respironics, NV, USA) and introduced to a Plexiglas chamber box (130 x 200 mm, 100 mm height) in which the mice were placed. Twenty-four h after the last OA challenge, mice were sacrificed by exsanguination from abdominal aorta under urethane (1.6 g/kg, *i*.*p*.; Sigma, St. Louis, MO) anesthesia.

### RNA extraction

Both left and right main bronchi were isolated, and the epithelia, capillary vessels and connective tissues were removed as much as possible (S1 Fig) by gently rubbing with sharp tweezers under a stereomicroscope [21]. Total RNA was extracted using Trizol reagent (Invitrogen, Karlsruhe, Germany) according to the manufacturer’s protocol. RNA purity and integrity were evaluated by ND-1000 Spectrophotometer (Thermo Fisher Scientific, Waltham, MA) and Agilent 2100 Bioanalyzer (Agilent Technologies, Santa Clara, CA).

### Microarray analysis

RNA labeling and hybridization were performed by using the Agilent One-Color Microarray-Based Gene Expression Analysis protocol (v6.5, Agilent Technologies). Briefly, total RNA (100 ng) of each sample was linearly amplified and labeled with Cy3-dCTP. The labeled cRNAs were purified using RNAeasy Mini Kit (Qiagen, Valencia, CA). The concentration and specific activity of the labeled cRNAs (pmol Cy3/μg cRNA) were measured by NanoDrop ND-1000 (Thermo Fisher Scientific Inc.). Each labeled cRNA (600 ng) was fragmented by adding 5 μL 10 x blocking agent and 1 μL of 25 x fragmentation buffer, and then heated at 60°C for 30 min. Finally 25 μL 2 x GE hybridization buffer was added to dilute the labeled cRNA. The hybridization solution (40 μL) was dispensed into the gasket slide and assembled to the Agilent SurePrint G3 Mouse GE 8X60K, v2 Microarrays (Agilent Technologies). The slides were incubated for 17 h at 65°C in an Agilent hybridization oven, and then washed at room temperature by using the Agilent One-Color Microarray-Based Gene Expression Analysis protocol (v6.5, Agilent Technologies). The hybridized array was immediately scanned with an Agilent Microarray Scanner D (Agilent Technologies).

The captured array images were analyzed using Agilent Feature Extraction Software (v11.0.1.1, Agilent Technologies). The raw data for same gene was then summarized automatically in Agilent Feature Extraction Protocol to generate raw data text file, providing expression data for each gene probed on the array. Array probes that have Flag A in samples were filtered out. Selected gProcessedSignal value was transformed by logarithm and normalized by quantile method. Statistical significance of the expression data was determined using fold change and local pooled error (LPE) test in which the null hypothesis was that no difference exists between the groups. Hierarchical cluster analysis was performed using complete linkage and Euclidean distance as a measure of similarity. Gene Ontology (GO) functional enrichment analysis for the differentially expressed genes was performed using Gene Set Enrichment Analysis software (http://software.broadinstitute.org/gsea/index.jsp). The gene sets were separated according to the GO terms for biological processes, cellular components, and molecular functions. Pathway analysis was performed using GeneCodis tools (http://genecodis.cnb.csic.es) based on the Kyoto Encyclopedia of Genes and Genomes (KEGG: https://www.kegg.jp) pathway database. All data analysis and visualization of differentially expressed genes was conducted using R 3.0.2 (www.r-project.org).

### Quantitative RT-PCR analyses

Expression levels of mRNA transcripts were determined by quantitative RT-PCR analysis. In brief, reverse transcription reactions were performed using a cDNA synthesis kit (RR037A: TaKaRa, Shiga, Japan) according to the manufacturer’s instructions. The cDNA products from each sample were then subjected to real-time PCR analyses using StepOne^™^ real-time PCR system (Applied Biosystems, Foster City, CA) with Fast SYBR Green Master Mix (Applied Biosystems) according to the manufacturer’s instructions. The reactions were incubated in a 48-well optical plate at 95°C for 20 seconds, following by 43 cycles of 95°C for 3 seconds and 60°C for 30 seconds. The PCR primer sets used are shown in Table 1, which were designed from published sequences.

**Table 1:** Primer sequences for RT-PCR used in the present study.

Gene name	RefSeq Accession		Sequence	Amplicon size
mouse Pla2g4c	NM_001168504	Sense	5’-GGACCGTTGCGTTTTTGTGA-3’	150 bp
		Antisense	5’-GCAAAACCAGCATCCACCAG-3’	
mouse Cyp4f18	NM_024444	Sense	5’-CTTCAGGGATGCATGCTGCT-3’	124 bp
		Antisense	5’-GAAGACTGTGTCCTTGGGGG-3’	
mouse Alox12e	NM_145684	Sense	5’-CTGAGGTTGGACTGCTTGGA-3’	124 bp
		Antisense	5’-TGTGTAGATGCGTGCTGACC-3’	
mouse Ptgs2	NM_011198	Sense	5’-CCGTGGGGAATGTATGAGCA-3’	128 bp
		Antisense	5’-GGGTGGGCTTCAGCAGTAAT-3’	
mouse Hpgds	NM_019455	Sense	5’-TTCCCATGGGCAGAGAAAGA-3’	143 bp
		Antisense	5’-GCCCAGGTTACATAATTGCCT-3’	
mouse Cyp2e1	NM_021282	Sense	5’-CGAGGGGACATTCCTGTGTT-3’	140 bp
		Antisense	5’-CGGGCCTCATTACCCTGTTT-3’	
mouse Cyp4a10	NM_010011	Sense	5’-ACCTCCACAGGCAATGGCTA-3’	150 bp
		Antisense	5’-ATCTAGGAAAGGCACTTGGGAAG-3’	
mouse Cyp4a12	NM_172306, NM_177406	Sense	5’-CCTTACACGGAAATCATGGCAG-3’	112 bp
		Antisense	5’-AGGTCATCAAGGTGATGTGTTGGA-3’	
mouse Gapdh	NM_008084	Sense	5’-CCTCGTCCCGTAGACAAAATG-3’	100 bp
		Antisense	5’-TCTCCACTTTGCCACTGCAA-3’	

### Western blot analyses

Protein samples of the BSM tissues were subjected to 15% sodium dodecyl sulfate-polyacrylamide gel electrophoresis (SDS-PAGE) and the proteins were then electrophoretically transferred to a polyvinylidene fluoride (PVDF) membrane. After blocking with EzBlock Chemi (Atto, Co., Tokyo, Japan), the PVDF membrane was incubated with the primary antibody. The primary antibodies used in the present study were polyclonal rabbit anti-hematopoietic prostaglandin D synthase antibody (HPGDS; 1:200 dilution; Item No. 160013; Cayman Chemical; Ann Arbore, MI). Then the membrane was incubated with horseradish peroxidase-conjugated donkey anti-rabbit IgG (1:2,500 dilution; Santa Cruz Biotechnology, Inc.; Santa Cruz, CA), detected using EzWestBlue (Atto, Co.) and analyzed by a densitometry system. Detection of house-keeping gene was also performed on the same membrane by using monoclonal mouse anti-GAPDH (1:10,000 dilution; Santa Cruz Biotechnology, Inc.).

### Assessment of prostaglandin D_2_ (PGD_2_) levels in BAL fluids

After the exsanguinations, the chest of each animal was opened and a 20-gauge blunt needle was tied into the proximal trachea. Bronchoalveolar lavage (BAL) fluid was obtained by intratracheal instillation of 1 mL/animal of phosphate-buffered saline (PBS; pH 7.5, room temperature) into the lung while it was kept located within the thoracic cavity. The lavage was reinfused into the lung twice before final collection. The BAL fluids were centrifuged at 500 g, and the resultant supernatants were stored at -80°C until use. The levels of PGD_2_ in BAL fluids were measured by a competitive PGD_2_ ELISA system (Item No. 512011: Cayman Chemical, Ann Arbor, MI) according to the manufacturer’s instructions.

### Lipidomic profiling by LC mass spectrometry

Lipid mediators were analyzed by LC and MS essentially as described previously [22, 23]. Briefly, BAL fluids were supplemented with a mix consisting of four deuterated internal standards (Cayman Chemical) and lipid metabolites isolated by solid phase extraction on an Oasis HLB column (Waters, Milford, MA). The extracted samples were evaporated, reconstituted in a small volume, and the eicosanoids were separated by reverse phase LC using an XBridge C18 column (Waters). The eicosanoids were analyzed by a triple quadrupole mass spectrometer (LCMS8040, Shimadzu, Kyoto, Japan) operated in the negative-ionization mode via multiple-reaction monitoring (MRM) using transitions that were optimized for selectivity and sensitivity. Quantitation was performed using calibration curves constructed for each compound, and recoveries were monitored using deuterated internal standards (15-HETE-d8, LTB_4_-d4, PGE_2_-d4, arachidonic acid-d8). Data analysis was performed using LabSolutions software (Shimadzu).

### Determination of bronchial smooth muscle (BSM) responsiveness

Mice were sacrificed by exsanguination from abdominal aorta under urethane (1.6 g/kg, *i*.*p*.) anesthesia and the airway tissues under the larynx to lungs were immediately removed. About 3 mm length of the left main bronchus (about 0.5 mm diameter) was isolated. The resultant tissue ring preparation was then suspended in a 5 mL-organ bath by two stainless-steel wires (0.2 mm diameter) passed through the lumen. For all tissues, one end was fixed to the bottom of the organ bath while the other was connected to a force-displacement transducer (TB-612T, Nihon Kohden) for the measurement of isometric force. A resting tension of 0.5 g was applied. The buffer solution contained modified Krebs-Henseleit solution with the following composition (mM); NaCl 118.0, KCl 4.7, CaCl_2_ 2.5, MgSO_4_ 1.2, NaHCO_3_ 25.0, KH_2_PO_4_ 1.2 and glucose 10.0. The buffer solution was maintained at 37°C and oxygenated with 95% O_2_-5% CO_2_. After the equilibration period, the tension studies were performed. In case of the high K^+^ depolarization studies, experiments were conducted in the presence of atropine (10^−6^ M).

### Data and statistical analyses

In the real-time PCR analyses, the comparative threshold cycle (C_T_) method was used for relative quantification of the mRNA transcripts. Differences in the C_T_ values (ΔC_T_) between the target gene and GAPDH were calculated to determine the relative expression levels, using the following formula: ΔΔC_T_ = (ΔC_T_ of the treated sample)–(ΔC_T_ of the control sample). The relative expression level between the samples was calculated according to the equation 2^-ΔΔCT^.

All the data were expressed as the mean with S.E. Statistical significance of difference was determined by unpaired Student’s *t*-test or two-way analysis of variance (ANOVA) with *post hoc* Bonferroni/Dunn (StatView for Macintosh ver. 5.0, SAS Institute, Inc., NC). A value of *P* < 0.05 was considered significant.

## Results

### Identification of differentially expressed genes in BSM tissues of the antigen-challenged mice

**Fig 1:**
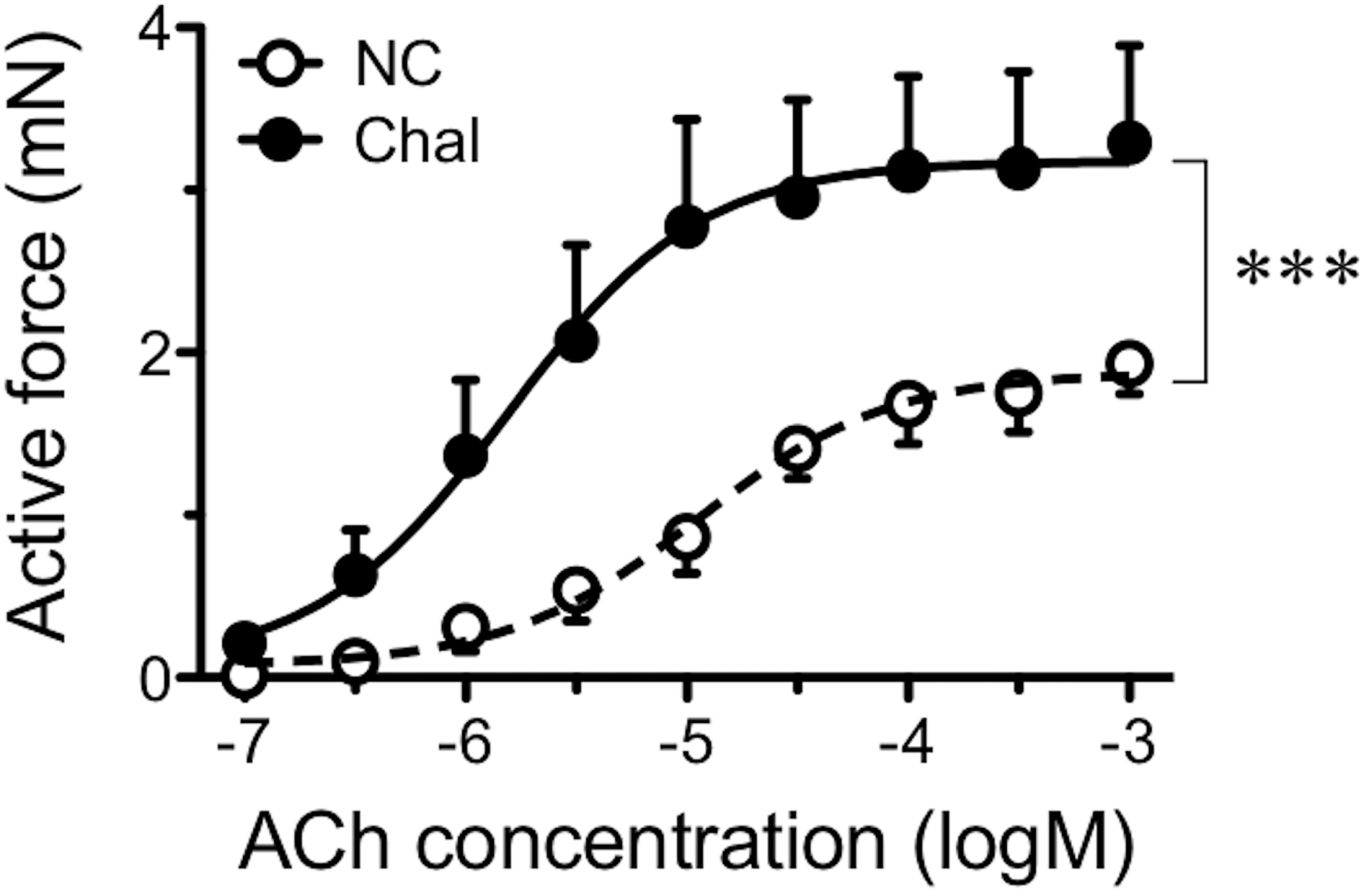
Change in the contractile responsiveness to acetylcholine (ACh) in bronchial smooth muscle tissues of a murine asthma model used in the present study. Male BALB/c mice were actively sensitized and repeatedly challenged with ovalbumin (OA) antigen. Twenty-four hours after the last OA challenge, the left main bronchi were isolated and the ACh responsiveness was measured as described in METHODS. Each point represents the mean ± SEM from 6 animals, respectively. NC: naive control, and Chal: repeatedly antigen-challenged groups. A significant difference was observed between the groups (*P* < 0.001 by two-way ANOVA with *post hoc* Bonferroni/Dunn test).

As shown in Fig 1, the contractile responsiveness to acetylcholine (ACh) was significantly augmented in bronchial smooth muscle (BSM) tissues isolated from the repeatedly antigen-challenged mice. In the present study, total RNA was isolated from BSM tissues of the diseased animals and was used for a DNA microarray analysis.

**Fig 2:**
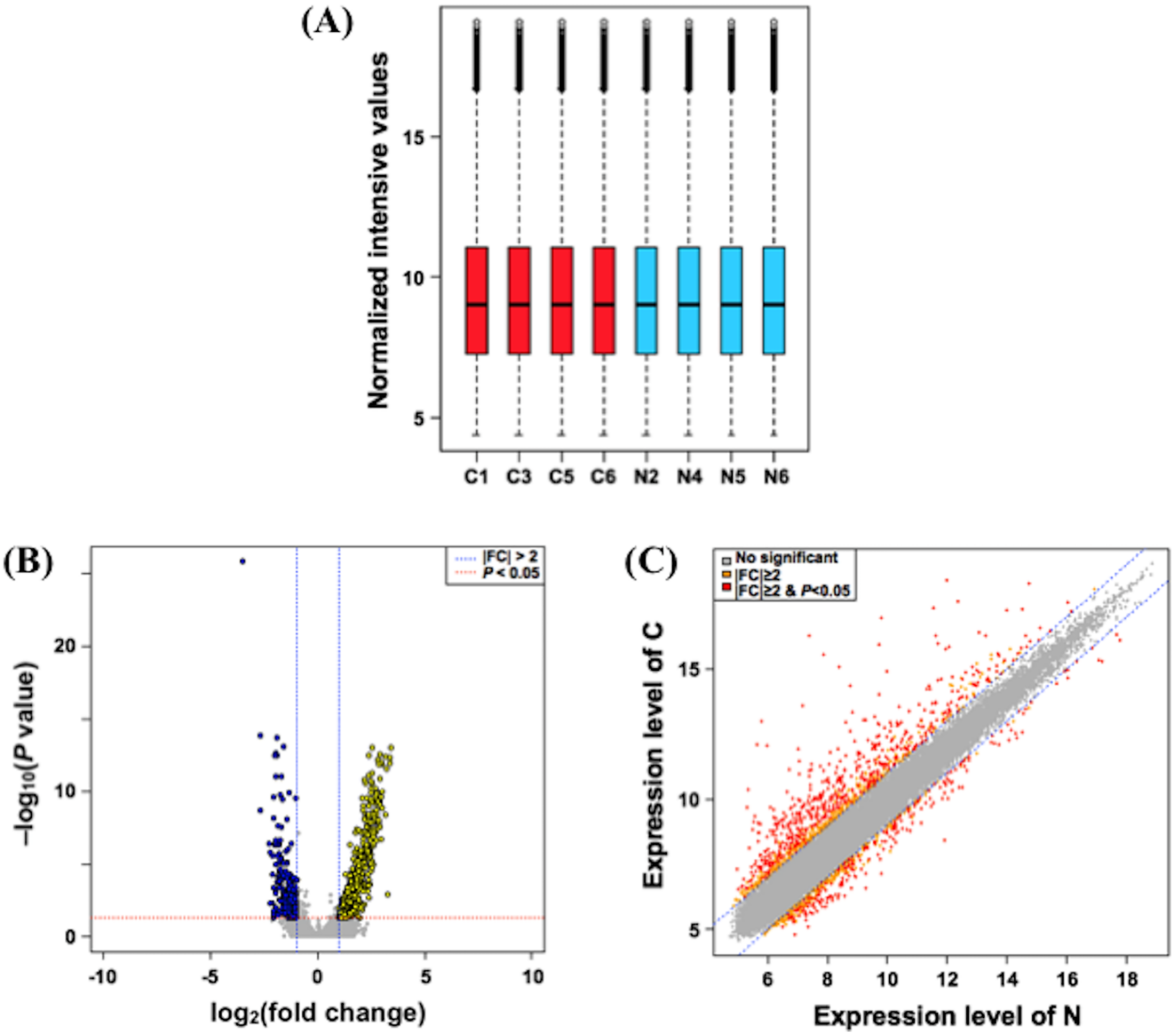
Expression profiles of genes in bronchial smooth muscle tissues of the repeatedly antigen-challenged (C) and normal control (N) mice. Total RNA sample of each mouse (4 animals, respectively) was subjected to the microarray analysis as described in METHODS. (A) Box-whisker plots of genes show the distribution of intensities from all samples. (B) Volcano plots show variation in gene expression. The vertical lines correspond to 2.0-fold up- and down-regulations. The horizontal line represents a *P*-value of 0.05. (C) Scatter plots show variation in gene expression. FC: fold change.

**Fig 3:**
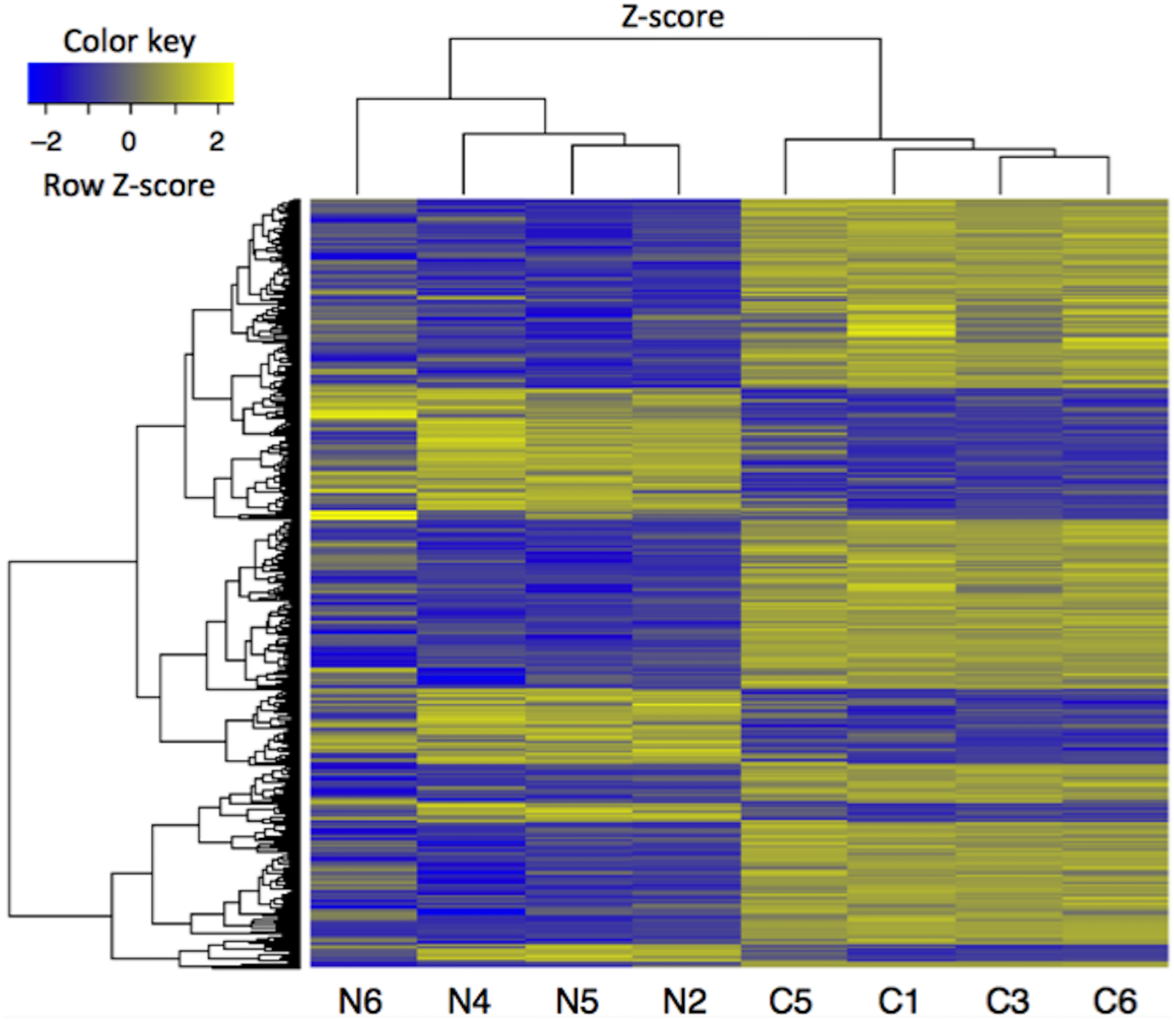
Hierarchical cluster analysis of differentially expressed genes (|fold change| ≥ 2 and adjusted *P*-value < 0.05) in bronchial smooth muscle tissues of the antigen-challenged mice. Total RNA sample of each mouse (4 animals, respectively) was subjected to the microarray analysis, and hierarchical cluster analysis of differentially expressed genes was performed. Each row represents a differentially expressed gene and each column represents an individual mouse. N and C represent the normal control and the repeatedly antigen-challenged groups, respectively. Each group contains four different animals. Colors represent fold change in each individual, with yellow indicating up-regulated genes and blue indicating down-regulated genes with respect to the average of the normal control animals.

As shown in Fig 2A, box-whisker plotting showed similar distribution of intensities among the samples used, suggesting that the array experiment was performed under an appropriate condition. Valiations of gene expression among the specimens were shown by volcano plotting (Fig 2B) and scatter plotting (Fig 2C). Of the 56,605 probe sets represented on the Agilent SurePrint G3 Mouse GE 8X60K v2 gene chip, 770 probe sets were differentially expressed in BSM tissues of the antigen-challenged mice as compared with those of control animals (|fold change| ≥ 2 and adjusted *P*-value < 0.05; N = 4, respectively). Among them, 557 were up-regulated and 213 were down-regulated. An unsupervised hierarchical clustering analysis of the differentially expressed genes showed a distinct separation between the antigen-challenged (C) and normal control animals (N) (Fig 3). The complete data set is publicly available in the Gene Expression Omnibus (GEO) public repository (http://www.ncbi.nlm.nih.gov/geo/) (Accession No. GSE116504) in a format that complies with the Minimal Information About a Microarray Experiment guidelines.

### Gene ontology analysis and KEGG pathway enrichment analysis

**Fig 4:**
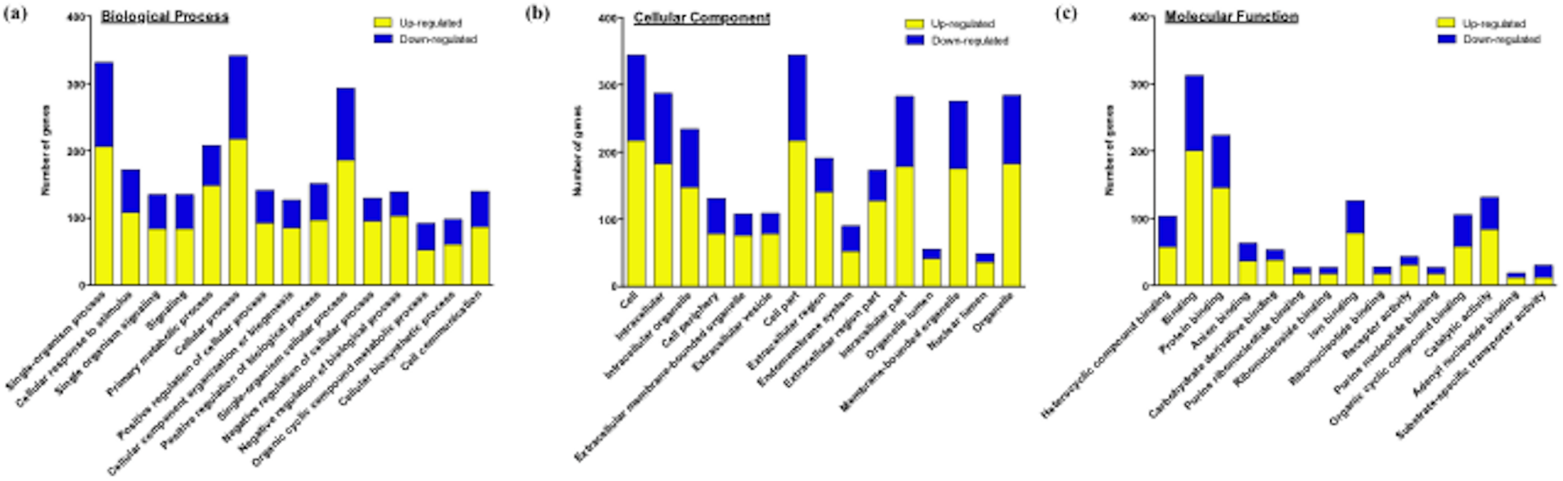
Gene ontology (GO) enrichment analysis of differentially expressed genes in bronchial smooth muscle tissues of the antigen-challenged mice. Total RNA sample of each mouse (4 animals, respectively) was subjected to the microarray analysis, and GO enrichment analysis of differentially expressed genes was performed. Top 15 significant GO terms for biological process (A), cellular component (B) and molecular function (C) are shown (adjusted *p*-value < 0.001 by Bonferroni). Each column represents the number of up- (yellow) and down-regulated (blue) genes in the indicated GO term.

**Fig 5:**
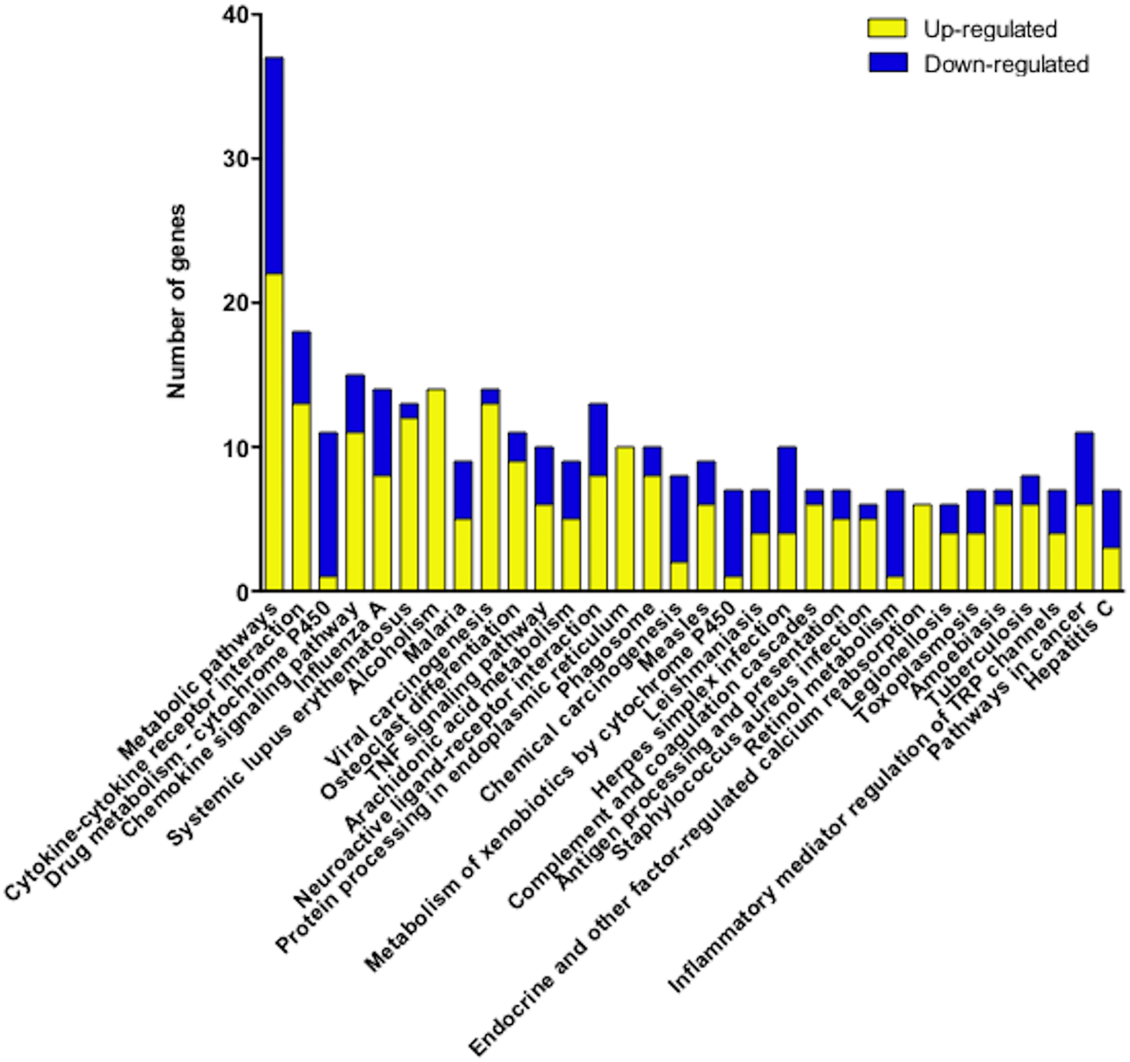
Kyoto Encyclopedia of Genes and Genomes (KEGG) pathway analysis of differentially expressed genes in bronchial smooth muscle tissues of the antigen-challenged mice. Significantly affected pathways are shown (adjusted *P*-value < 0.05 by Bonferroni). Total RNA sample of each mouse (4 animals, respectively) was subjected to the microarray analysis, and KEGG pathway analysis of differentially expressed genes was performed. Each column represents the number of up- (yellow) and down-regulated (blue) genes in the indicated pathway.

**Fig 6:**
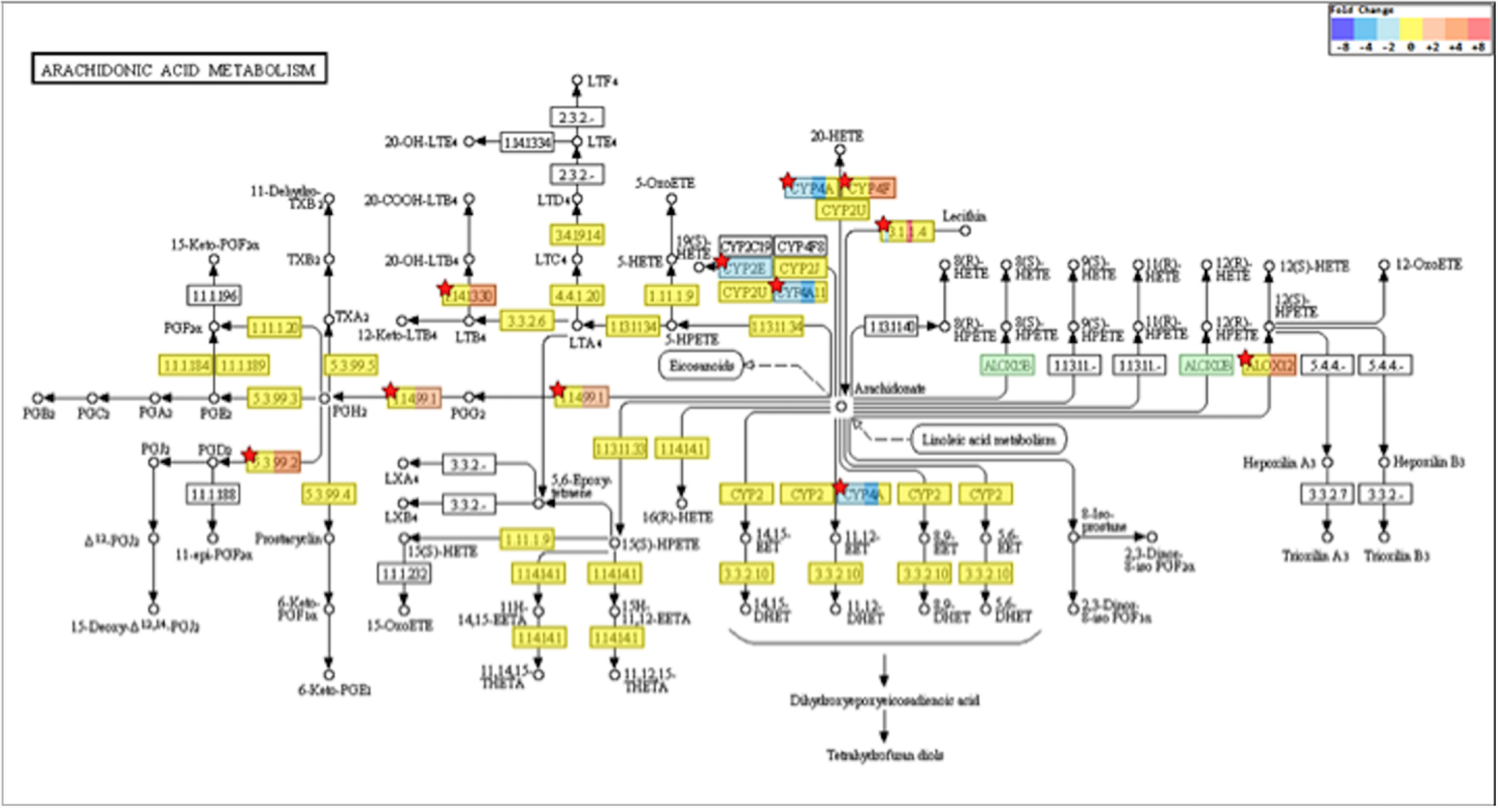
Change in the arachidonic acid metabolism pathway (KEGG map ID: mmu00590) based on the differentially expressed genes in bronchial smooth muscle tissues of the antigen-challenged mice. Total RNA sample of each mouse (4 animals, respectively) was subjected to the microarray analysis, and Kyoto Encyclopedia of Genes and Genomes pathway analysis of differentially expressed genes was performed. Enzymatic reactions are marked by arrows. Fold change values of differentially expressed genes are shown in colors. Different colors in a box for the same module indicate various genes with differing expression. White boxes of pathway map indicate modules that are not relevant to the mouse. Green color boxes of pathway map indicate modules that are not mapped (the gene is in the pathway map but its expression was not shown in the present study). The pathway modules containing differentially expressed genes with statistical significance are marked with red stars.

Gene ontology analysis of the differentially expressed genes identified a number of different processes with statistical significance. Fig 4 shows the top 15 gene ontology terms of biological processes (Fig 4A), cellular components (Fig 4B), and molecular function (Fig 4C). The KEGG pathway analysis suggested that 69 pathways were significantly correlated with the differentially expressed genes (adjusted *P*-value < 0.05 by FDR). These included pathways reported to be associated with asthma, such as Cytokine-cytokine receptor interaction (Map ID: mmu04060: adjusted *P*-value = 5.5E-11), Chemokine signaling pathway (Map ID: mmu04062: adjusted *P*-value = 1.0E-8), TNF signaling pathway (Map ID: mmu04668: adjusted *P*-value = 1.9E-6), and Asthma (Map ID: mmu05310: adjusted *P*-value = 0.040). When the Bonferroni correction was applied to the data, 32 pathways were suggested as highly significant pathways (adjusted *P*-value < 0.05 by Bonferroni: Fig 5). Among them, we focused on Arachidonic acid (AA) metabolism pathway (Map ID: mmu00590, Fig 6) in the present study. The AA metabolism pathway comprised 9 differentially expressed genes: 5 of them were significantly up-regulated and 4 of them were significantly down-regulated in BSM tissues of the antigen-challenged mice (adjusted *P*-value < 0.05 by Bonferroni, Table 2).

**Table 2:** Differentially expressed genes included in the arachidonic acid metabolism pathway (KEGG Map ID: mmu00590) in bronchial smooth muscles of mice with allergic asthma.

Probe ID	Gene_Symbol	Gene_ID	RefSeq Accession	Fold change	Adjusted *P*-value
A_52_P17207	Pla2g4c	232889	NM_001168504	9.890340	0
A_55_P2487484	Cyp4f18	72054	NM_024444	6.538004	2.75667E-10
A_51_P471659	Alox12e	11685	NM_145684	4.582966	1.13732E-06
A_51_P254855	Ptgs2	19225	NM_011198	3.418893	0.000107618
A_52_P536796	Hpgds	54486	NM_019455	3.251222	0.004657938
A_51_P283456	Cyp2e1	13106	NM_021282	-2.780193	2.87373E-05
A_55_P2092030	Cyp4a10	13117	NM_010011	-3.643348	2.38762E-08
A_55_P2152607	Cyp4a12b	13118	NM_172306	-3.908007	9.61085E-12
A_55_P2043083	Cyp4a12a	277753	NM_177406	-4.040550	3.26138E-13

### Validation of differentially expressed genes by RT-qPCR

**Fig 7:**
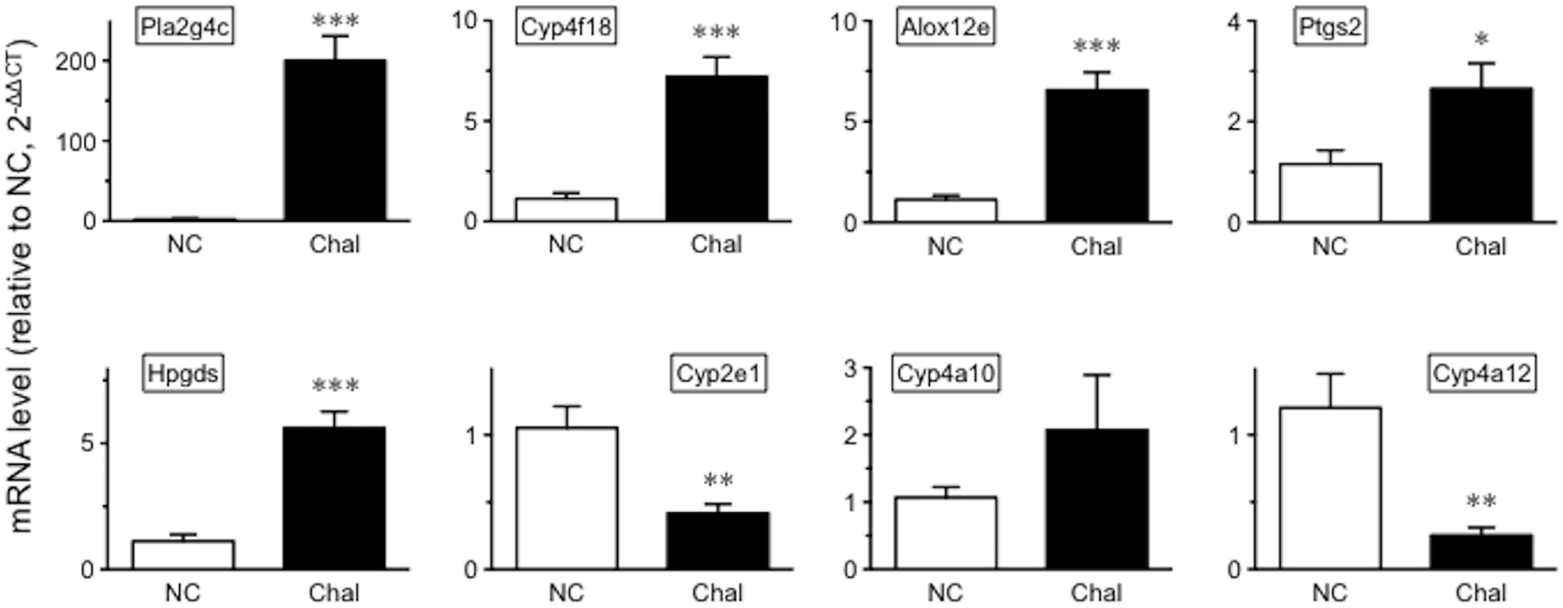
Quantitative RT-PCR validation of differentially expressed genes included in the arachidonic acid metabolism pathway in bronchial smooth muscle tissues of the antigen-challenged mice. Each column represents the mean ± SEM from 6 animals in duplicate, respectively. NC: naive control, and Chal: repeatedly antigen-challenged groups. *P < 0.05, **P < 0.01 and ***P < 0.001 by unpaired Student’s *t*-test.

In order to validate the microarray data, differentially expressed mRNA transcripts included in the AA pathway were further analyzed by real-time RT-qPCR (Fig 7). Since the PCR primer design did not allow us to distinguish between *Cyp4a12a* and *Cyp4a12b*, the sum of these transcripts were measured and referred to as *Cyp4a12*. With the exception of *Cyp4a10*, all mRNA transcripts examined showed the concordant up- or down-regulation in BSM tissues of the antigen-challenged mice (Fig 7).

### Prostaglandin D_2_ level in bronchoalveolar lavage fluids

**Fig 8:**
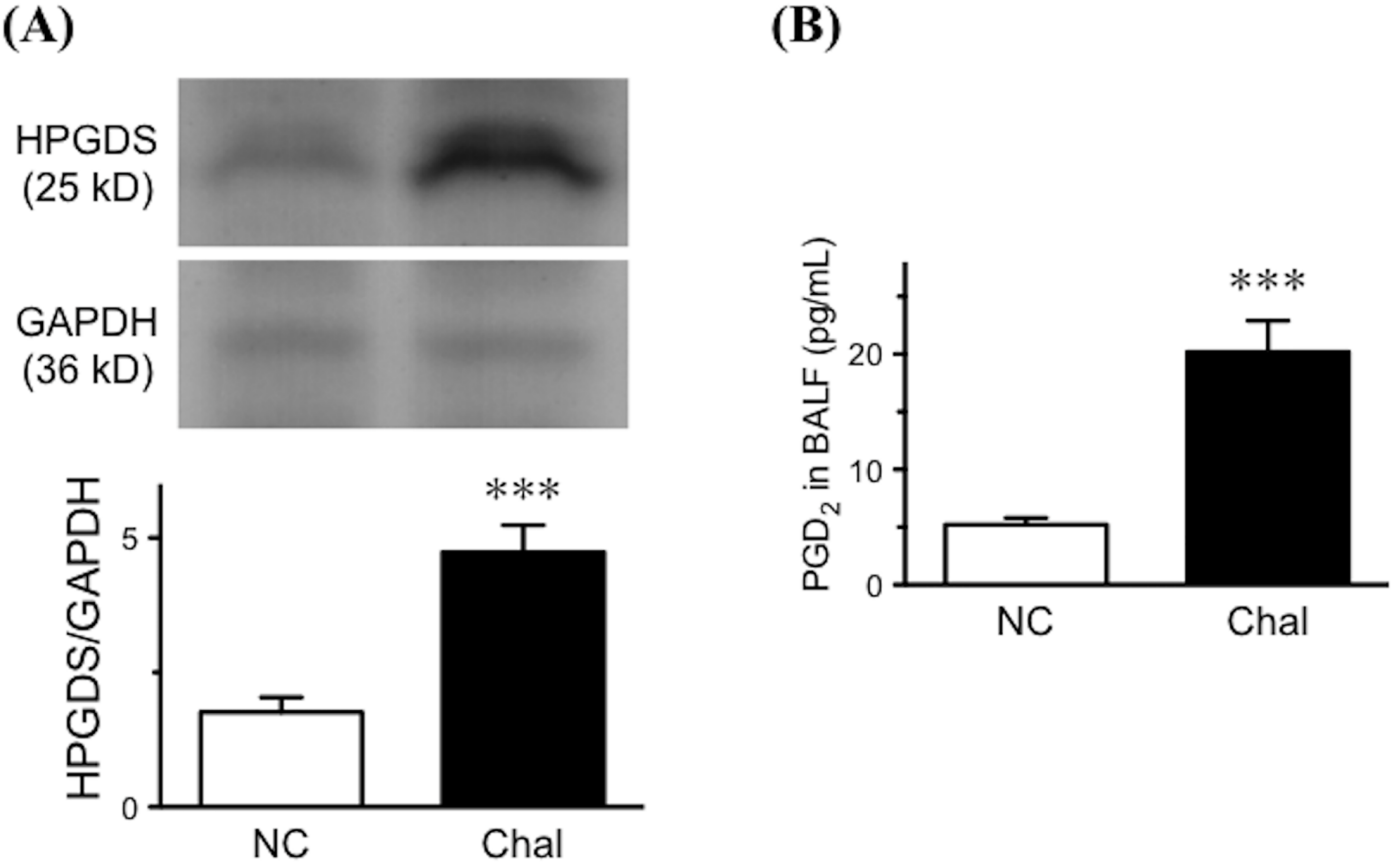
**(A) Change in the expression levels of hematopoietic prostaglandin D synthase (HPGDS) protein determined by immunoblottings.** (Upper panel) Representative blots for HPGDS and GAPDH. The bands analyzed by a densitometer and normalized by the intensity of corresponding GAPDH band, and the data are summarized in the lower panel. Results are presented as mean ± SEM from 5 animals in duplicate, respectively. ***P < 0.001 by unpaired Student’s *t*-test. **(B) Change in prostaglandin D**_**2**_ **(PGD**_**2**_**) level in bronchoalveolar lavage (BAL) fluids of the antigen-challenged mice.** Twenty-four hours after the last antigen challenge, BAL fluids were obtained, and PGD_2_ concentrations in BAL fluids were determined by enzyme-linked immunosorbent assay. Results are presented as mean ± SEM from 5 animals in triplicate, respectively. ***P < 0.001 by unpaired Student’s *t*-test.

The results of microarray and RT-qPCR analyses revealed that the expression of phospholipase A_2_ (*Pla2g4c*), cyclooxygenase-2 (*Ptgs2*) and prostaglandin D synthase (*Hpgds*) were significantly increased in BSM tissues of the antigen-challenged mice. Immunoblot analysis also revealed an up-regulation of HPGDS protein in BSM tissues of the diseased mice (Fig 8A). The findings also strongly suggest that the AA metabolism is largely shifted towards prostaglandin D_2_ (PGD_2_) production in BSM tissues of the murine asthma model. To determine the changes in AA metabolism in the airways, lipid mediators in bronchoalveolar lavage (BAL) fluids were measured using LC/MS/MS and ELISA.

**Fig 9:**
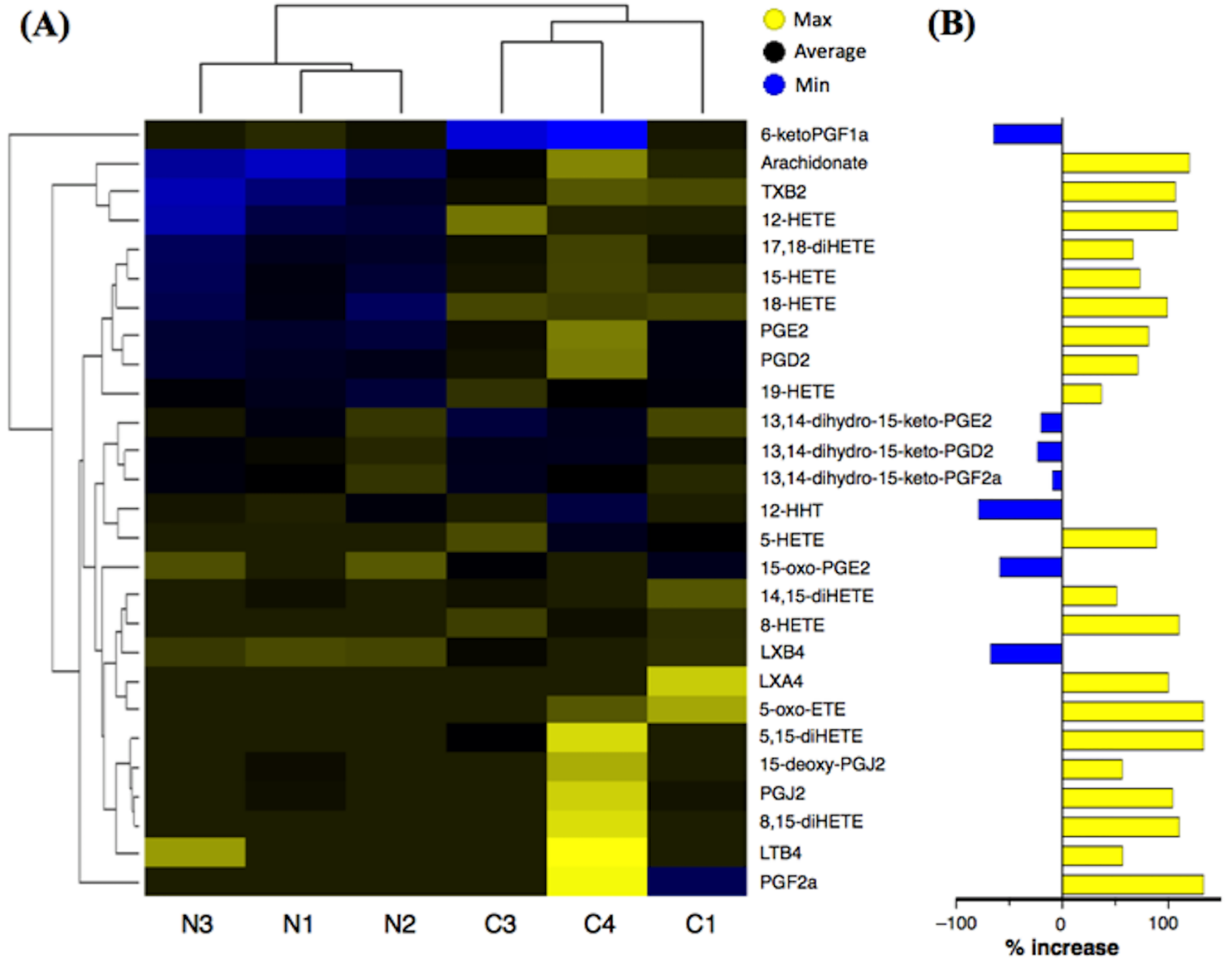
Profiling of changes in arachidonic acid metabolites in bronchoalveolar lavage (BAL) fluids of the antigen-challenged mice. Twenty-four hours after the last antigen challenge, BAL fluids were obtained from respective mice. The BAL fluid of each mouse (3 animals/group) was subjected to the LC/MS/MS analysis as described in METHODS. (A) Hierarchical cluster analysis of arachidonic acid metabolites measured. Each row represents a metabolite and each column represents an individual mouse. N and C represent the normal control and the repeatedly antigen-challenged groups, respectively. Each group contains three different animals. Colors represent fold change in each individual, with yellow indicating increased metabolites and blue indicating decreased metabolites with respect to the average of the normal control animals. (B) Summary of % changes in the metabolite levels in BAL fluids of antigen-challenged mice. Yellow columns indicate % increases and blue columns indicate % decreases of the indicated arachidonic acid metabolites.

In BAL fluids of the mice, a total of 78 lipid species were able to measure by using LC/MS/MS approach. Among them, a total of 27 AA-related metabolites were consistently detected in samples (Fig 9). The level of AA itself in BAL fluids of the antigen-challenged mice (284.5 ± 81.7 pg/mL) was significantly higher than that of control animals (22.2 ± 7.2 pg/mL, *P* < 0.05 by unpaired Student’s *t*-test). In the repeatedly antigen-challenged group, most of the AA-related metabolites including PGD_2_ were increased (Fig 9B), but the levels of 6-keto-prostaglandin F_1_alpha (6-keto-PGF_1_alpha; 952.1 ± 44.2 pg/mL in control *versus* 60.2 ± 27.0 pg/mL in challenged mice, *P* < 0.001 by unpaired Student’s *t*-test) and 12S-hydroxy-5Z,8E,10E-heptadecatrienoic acid (12-HHT; 6.4 ± 0.9 pg/mL in control *versus* 0.4 ± 0.4 pg/mL in challenged mice, P < 0.001 by unpaired Student’s *t*-test) were significantly decreased. The levels of PGD_2_ in BAL fluids were further determined using an ELISA system. As shown in Fig 8B, the PGD_2_ level in BAL fluids of the antigen-challenged mice was significantly higher than that of control animals (*P* < 0.001 by unpaired Student’s *t*-test).

### Effects of prostaglandin D_2_ on BSM function

**Fig 10:**
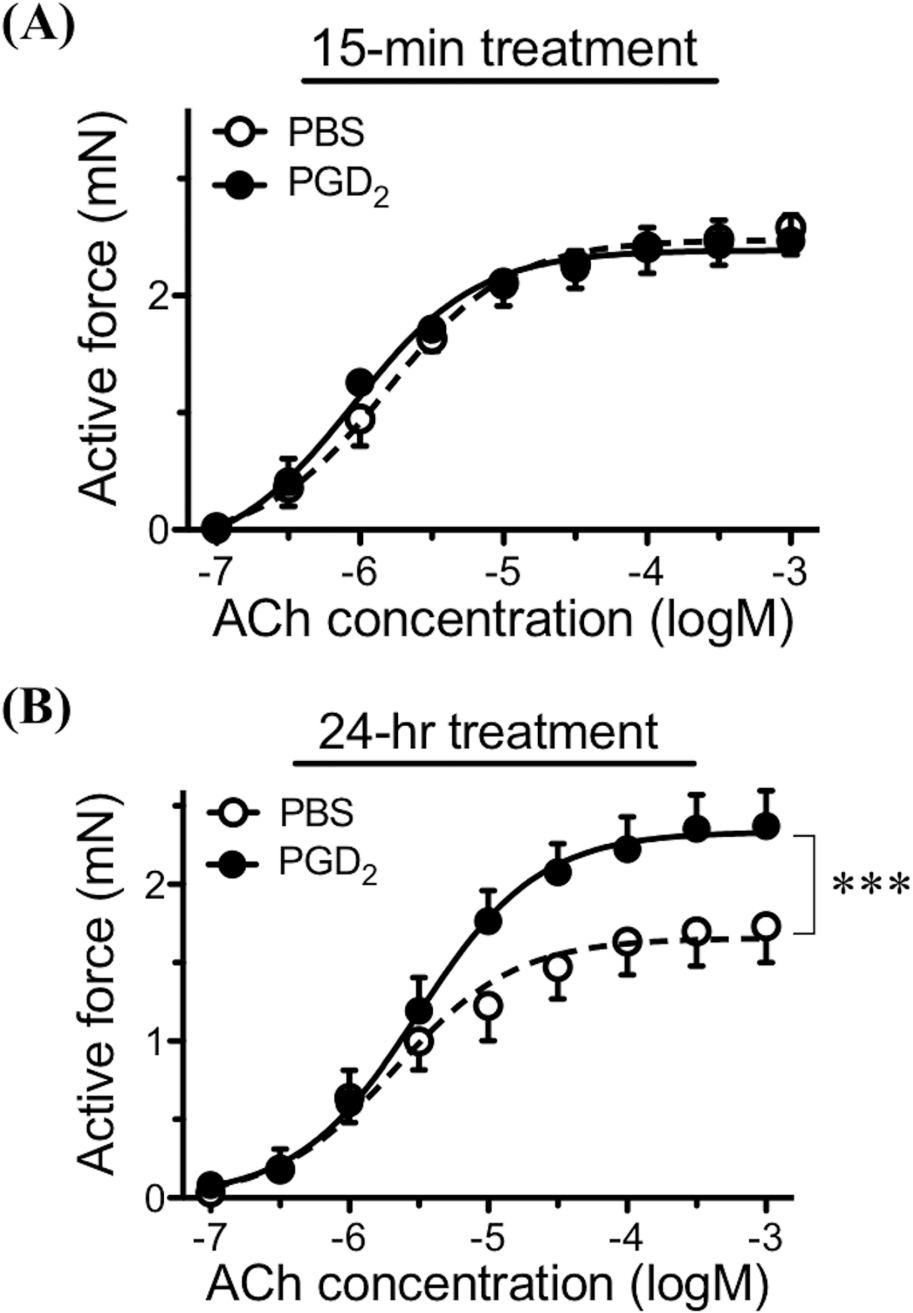
Effect of prostaglandin D_2_ (PGD_2_) on bronchial smooth muscle (BSM) contractility in naive mice. The BSM tissues isolated from naive BALB/c mice (6 animals) were incubated with PGD_2_ (10^−5^ M: closed circles) or its vehicle (PBS: open circles) for 15 minutes (A) or 24 hours (B). The BSM responsiveness to cumulatively applied acetylcholine (ACh) was measured as described in METHODS. The ACh concentration-response curve was significantly shifted upward when the BSMs were incubated with PGD_2_ for 24 hours (B: ***P < 0.001 by one-way ANOVA with *post hoc* Dunnett).

To determine the role of prostaglandin D_2_ (PGD_2_) on the AHR, effects of PGD_2_ on the isometric tension of smooth muscles were examined in BSM tissues isolated from naive control mice. Application of PGD_2_ (10^−9^~10^−5^ M) had no effect on basal tone of the BSM tissues (data not shown). When the BSM tissues were pre-contracted with 10^−5^ M ACh (about a half-maximal contraction) or 60 mM K^+^ (in the presence of 10^−6^ M atropine), cumulatively applied PGD_2_ (10^−9^~10^−5^ M) showed a complicated response. A transient inhibitory effect was observed at the PGD_2_ concentration of 10^−6^ M: this was followed by an enhancing effect by the application of 10^−5^ M of PGD_2_ (data not shown), indicating that a higher concentration of PGD_2_, at least, could augment the BSM contraction in naive control animals. So in the present study, effects of pre-treatment with PGD_2_ (10^−5^ M) on the BSM responsiveness to ACh were determined. As shown in Fig 10A, a 15-min treatment with PGD_2_ had no effect on the BSM responsiveness to ACh. However, the ACh concentration-response curve was significantly shifted upward when the BSM tissues were incubated with PGD_2_ for 24 hours (Fig 10B).

## Discussion

Augmented contractility of airway smooth muscles is one of the causes of the AHR in asthmatics [1–4, 24]. However, the mechanism of the altered properties of airway smooth muscle cells is not fully understood now. In the present study, we focused on BSM tissues of the antigen-challenged mice that have both *in vivo* AHR [20] and hyper-contractility of the isolated BSM tissues (Fig 1). To screen differentially expressed genes of the diseased BSM tissues, a DNA microarray analysis was applied using total RNA extracted from the BSM tissues. Of the 56,605 probe sets represented on the gene chip used, 557 were up-regulated and 213 were down-regulated (see Results section), indicating that gene expression is abundantly changed in the BSM tissues of asthma. The KEGG pathway analysis of the microarray data revealed a significant change in the AA metabolism pathway in BSM tissues of the antigen-challenged mice (Fig 5). In particular, an augmentation of PLA_2_ group 4c (*Pla2g4c*)/COX-2 (*Ptgs2*)/PGD_2_ synthase 2 (*Hpgds*) cascade was strongly suggested (Fig 6). Expression of these genes in smooth muscle cells of the airways has also been shown by the published gene expression data (GEO accession number GSE45723) [25] and the current study (S2 Fig).

Eicosanoids, including prostaglandins, thromboxanes and leukotrienes, are important signaling molecules that have been implicated in various pathological processes including asthma [26–28]. Their precursor AA is freed from cell membrane phospholipid by the action of PLA_2_ family of enzymes. In mice, 20 genes of the PLA_2_ family (EC: 3.1.1.4) are listed in the KEGG AA metabolism pathway (Map ID: mmu00590). Among them, a total of 12 genes, *Pla2g2c*, *Pla2g2d*, *Pla2g4a*, *Pla2g5*, *Pla2g4b*, *Pla2g16*, *Pla2g4c*, *Pla2g3*, *Pla2g2e*, *Pla2g6*, *Pla2g12a*, and *Plb1*, were consistently expressed in the BSM tissues by the current microarray analysis. Among the 12 genes expressed, a dramatic increase in *Pla2g4c* (PLA_2_ group 4c) was observed in BSM tissues of the OA-challenged mice as compared to those of control animals (Table 2 and Fig 7). The results might be consistent with previous report where an increase in the expression of *Pla2g4c* was demonstrated in lungs of a murine asthma model induced by *Aspergillus fumigatus* [29]. It is thus possible that increased expression of *Pla2g4c* is a ubiquitous event in the airways of allergic asthma. PLA_2_ group 4c, also named as cytosolic PLA_2_gamma (cPLA_2_gamma), was first identified by Pickard and colleagues [30], and is a Ca^2+^-independent enzyme on its PLA_2_ activity [31, 32]. Cells overexpressing cPLA_2_gamma could cause an increase in AA release [31, 33, 34]. Elevated levels of AA in BAL fluids were reported in asthmatics after inhaled antigen challenge [35]. Thus, up-regulation of PLA_2_ group 4c might be responsible for the increased AA level in the airways (Fig 9) and the subsequent increase in eicosanoids that are implicated in asthma pathology [26–28].

PGD_2_ is an acidic lipid mediator derived from the metabolism of AA by the action of cyclooxygenases, COX-1 (*Ptgs1*) and COX-2 (*Ptgs2*), and downstream PGD_2_ synthases, lipocalin-type PGD synthase (*Ptgds*) and hematopoietic PGD synthase (*Hpgds*). Current microarray analyses revealed that these genes were expressed in BSM tissues of the mice. Among them, the mRNA expression levels of *Ptgs2* and *Hpgds* were significantly increased in BSM tissues of the OA-challenged animals (Fig 6 and Table 2). Likewise, an increased expression of COX-2 has been demonstrated in lungs of guinea pig asthma model [36] and airway smooth muscles of patients with asthma [37]. Cytokine stimulation could cause an induction of COX-2 in human airway smooth muscle cells [38, 39]. An up-regulation of HPGDS has also been reported in airway structural cells of asthmatics [40]. It is thus possible that the AA metabolism might largely shift towards PGD_2_ production in the airways of asthma. Indeed, a significant increase in PGD_2_ was also observed in BAL fluids of the OA-challenged mice (Figs 8 and 9).

To determine the role of PGD_2_ on smooth muscle function of the airways, the BSMs isolated from naive control mice were treated with PGD_2_. Reportedly, PGD_2_ has an ability to cause contraction of the isolated airway smooth muscles in guinea pigs [41, 42], rabbits [43] and dogs [44]. The contraction seems to be mediated partly by stimulating cholinergic neurotransmission [41, 44]. However, the current organ bath studies revealed that application of PGD_2_ did not affect on baseline tension of the mouse BSMs (see Results), indicating that there is species differences in the effect of PGD_2_ on airway smooth muscle function. On the other hand, PGD_2_ might be capable of inducing BSM hyper-contractility: PGD_2_ augmented the sub-maximal contraction induced by ACh (see Results) and high-K^+^ depolarization (in the presence of 10^−6^ M atropine: data not shown). Moreover, the 24-h incubation with PGD_2_ caused a BSM hyperresponsiveness to ACh (Fig 10B), as if that was observed at 24 h after the antigen challenge in the asthmatic animals (Fig 1). These findings suggest that the increased PGD_2_ level in the airways (Figs 8 and 9) is one of the causes of the antigen-induced BSM hyperresponsiveness.

In the present study, although the BSM contractility was significantly augmented in the antigen-challenged mice (Fig 1), no significant change in the expression levels of genes related to the smooth muscle contraction, such as myosins (*Myh11*, *Myl6*, *Myl9*), actins (*Acta2*, *Actg2*), myosin light chain kinases (MLCKs: *Mylk*, *Mylk2*~*4*), MLC phosphatases (MLCPs: *Ppp1r12a*, *Ppp1r12b*, *Ppp1r12c*, *Ppp1ca*, *Ppp1cb*, *Ppp1cc*), and caldesmon (*Cald1*), was detected by the current microarray analysis. In this animal model of asthma, an augmented RhoA-mediated Ca^2+^ sensitization of the BSM contraction is a cause of the BSM hyper-contractility [21]. It has also been demonstrated that an inhibition of negative regulation mediated by a microRNA (miRNA), miR-133a-3p, is the main cause of the up-regulation of RhoA protein [18, 45]. Similarly, miRNA regulation of airway smooth muscle function has also been reported [37, 46–50]. It is thus possible that changes in post-transcriptional rather than transcriptional modulations of the gene expression might be largely involved in the alteration of smooth muscle contractility of the diseased airways. PGD_2_ might cause such an epigenetic change in airway smooth muscle, resulting in its augmented contractility. Further studies are needed to make clear the exact role of PGD_2_ on airway smooth muscle function.

The current LC/MS/MS analysis also revealed a significant decrease in the levels of 6-keto-PGF_1_alpha, a stable metabolite of PGI_2_, in BAL fluids of the antigen-challenged mice (see Results), indicating that PGI_2_ production is decreased in the airways of asthma. PGI_2_ is produced from PGH_2_, an AA-derived COX metabolite, by the action of PGI_2_ synthase (*Ptgis*). Because PGI_2_ and its synthetic analogues have been suggested to induce a BSM relaxation [51] and to have an inhibitory effect of asthma including the AHR [51–53], the decreased PGI_2_ level in the airways might also be one of the causes of the AHR. Although the mechanism of decrease in PGI_2_ level is unclear now, its precursor AA was conversely increased in the diseased airways (see Results). No change in the expression of PGI_2_ synthase (*Ptgis*) was observed in the present microarray analysis (fold change: 1.05, adjusted *P*-value: *P* > 0.05). Similarly, the expression of thromboxane A synthase (*Tbxas1*) in the diseased BSM tissues was within control level (fold change: 1.66, adjusted *P*-value: *P* > 0.05). However, the LC/MS/MS approach also revealed a significant decrease in 12-HHT (see Results), whereas a significant increase in TXB_2_, a stable metabolite of TXA_2_, was observed in BAL fluids of the antigen-challenged mice (15.2 ± 7.6 pg/mL in control *versus* 99.2 ± 15.6 pg/mL in challenged mice, *P* < 0.01 by unpaired Student’s *t*-test). Both 12-HHT and TXA_2_ are generated from PGH_2_ by the action of *Tbxas1* [54]. It is thus important to note that changes in the levels of lipid mediators in asthma might not be explained simply by changes in the expression of related enzyme genes.

In conclusion, the current study demonstrated that the AA metabolism is largely shifted towards PGD_2_ production in BSM tissues of asthma. Increased PGD_2_ level in the airways might be one of the causes of airway smooth muscle hyper-contractility, that is a cause of the AHR in asthmatics. The PLA_2_ group 4c/COX-2/PGD_2_ synthase 2 cascade might be a potential therapeutic target for AHR in asthma, although some validation studies using human tissues should be required.

## Supporting information

S1 Fig Immunohistochemistry of αlpha-smooth muscle actin (alpha-SMA) in bronchial smooth muscle (BSM) tissues of mice. The main bronchi were isolated from mice as described in METHODS, and their cryostat sections (4 μm) were immunostained with anti-alpha-SMA antibody (1:500 dilution, overnight incubation: Cytoskeleton, Inc.). Typical immunofluorescent images of intact (A) and the mechanically epithelium-denudated BSM tissues (C) and their corresponding light images (B and D, respectively) are shown. e: epithelial layer, bm: basement membrane, and SM: smooth muscle layer.
(TIFF)

S2 Fig Expression of *Pla2g4c*, *Ptgs2* and *Hpgds* in the epithelium-denudated bronchial smooth muscle (BSM) tissues of mice determined by RT-PCR. cDNA samples of the BSMs were amplified using specific primer sets for mouse *Gapdh* (forward primer: 5’-CCTCGTCCCGTAGACAAAATG-3’, reverse primer: 5’-TCTCCACTTTGCCACTGCAA-3’), *Pla2g4c* (forward primer: 5’-GGACCGTTGCGTTTTTGTGA-3’, reverse primer: 5’-GCAAAACCAGCATCCACCAG-3’), *Ptgs2* (forward primer: 5’-CCGTGGGGAATGTATGAGCA-3’, reverse primer: 5’-GGGTGGGCTTCAGCAGTAAT-3’) and *Hpgds* (forward primer: 5’-TTCCCATGGGCAGAGAAAGA-3’, reverse primer: 5’-GCCCAGGTTACATAATTGCCT-3’), and detected by 2% agarose gel electrophoresis. Marker: M.W. markers (100 bp ladder).
(TIFF)

## Abbreviations

12-HHT: 12S-hydroxy-5Z,8E,10E-heptadecatrienoic acid
15-HETE: 15-hydroxy-5Z,8Z,11Z,13E- eicosatetraenoic acid
AA: arachidonic acid
ACh: acetylcholine
ACT: actin
AHR: airway hyperresponsiveness
ANOVA: analysis of variance
BAL: bronchoalveolar lavage
BSM: bronchial smooth muscle
CALD: caldesmon
COX: cyclooxygenase
cRNA: complementary RNA
C_T_: threshold cycle
dCTP: deoxycytidine triphosphate
DEG: differentially expressed gene
FDR: false discovery rate
GO: gene ontology
HPGDS: prostaglandin D_2_ synthase 2 (hematopoietic PGD synthase)
KEEG: Kyoto Encyclopedia of Genes and Genomes
LC/MS/MS: liquid chromatography/tandem mass spectrometry
LPE: local pooled error
LT: leukotriene
miRNA: microRNA
MYH: myosin heavy chain
MYL: myosin light chain
MYLK: myosin light chain kinase
OA: ovalbumin
PG: prostaglandin
PLA_2_: phospholipase A_2_
PLA2G4C: phospholipase A_2_ group 4c
PLB1: phospholipase B_1_
PPP1: protein phosphatase 1
PTGIS: prostaglandin I_2_ synthase
PTGS2: prostaglandin-endoperoxide synthase 2 (cyclooxygenase-2)
TBXAS1: thromboxane A synthase
TNF: tumor necrosis factor
