# Supplementary figures and images for: Augmented Pla2g4c/Ptgs2/Hpgds axis in bronchial smooth muscle tissues of experimental asthma
Source: PLoS One. 2018 Aug 30;13(8):e0202623. doi: 10.1371/journal.pone.0202623 (PMC6116991; doi:10.1371/journal.pone.0202623)

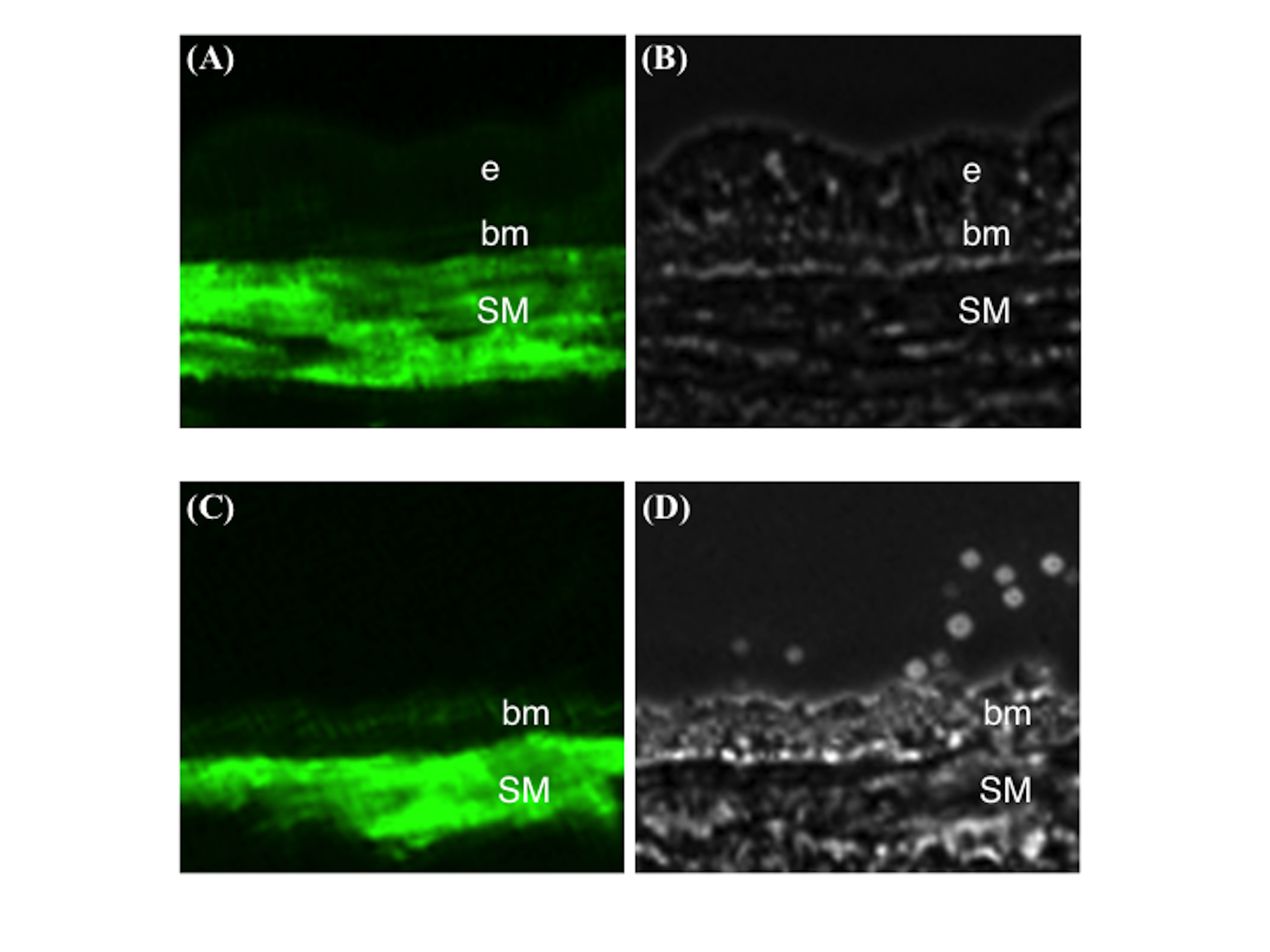

Supplement: S1 Fig — The main bronchi were isolated from mice as described in METHODS, and their cryostat sections (4 μm) were immunostained with anti-alpha-SMA antibody (1:500 dilution, overnight incubation: Cytoskeleton, Inc.). Typical immunofluorescent images of intact (A) and the mechanically epithelium-denudated BSM tissues (C) and their corresponding light images (B and D, respectively) are shown. e: epithelial layer, bm: basement membrane, and SM: smooth muscle layer. (TIFF) [file pone.0202623.s001.tiff]

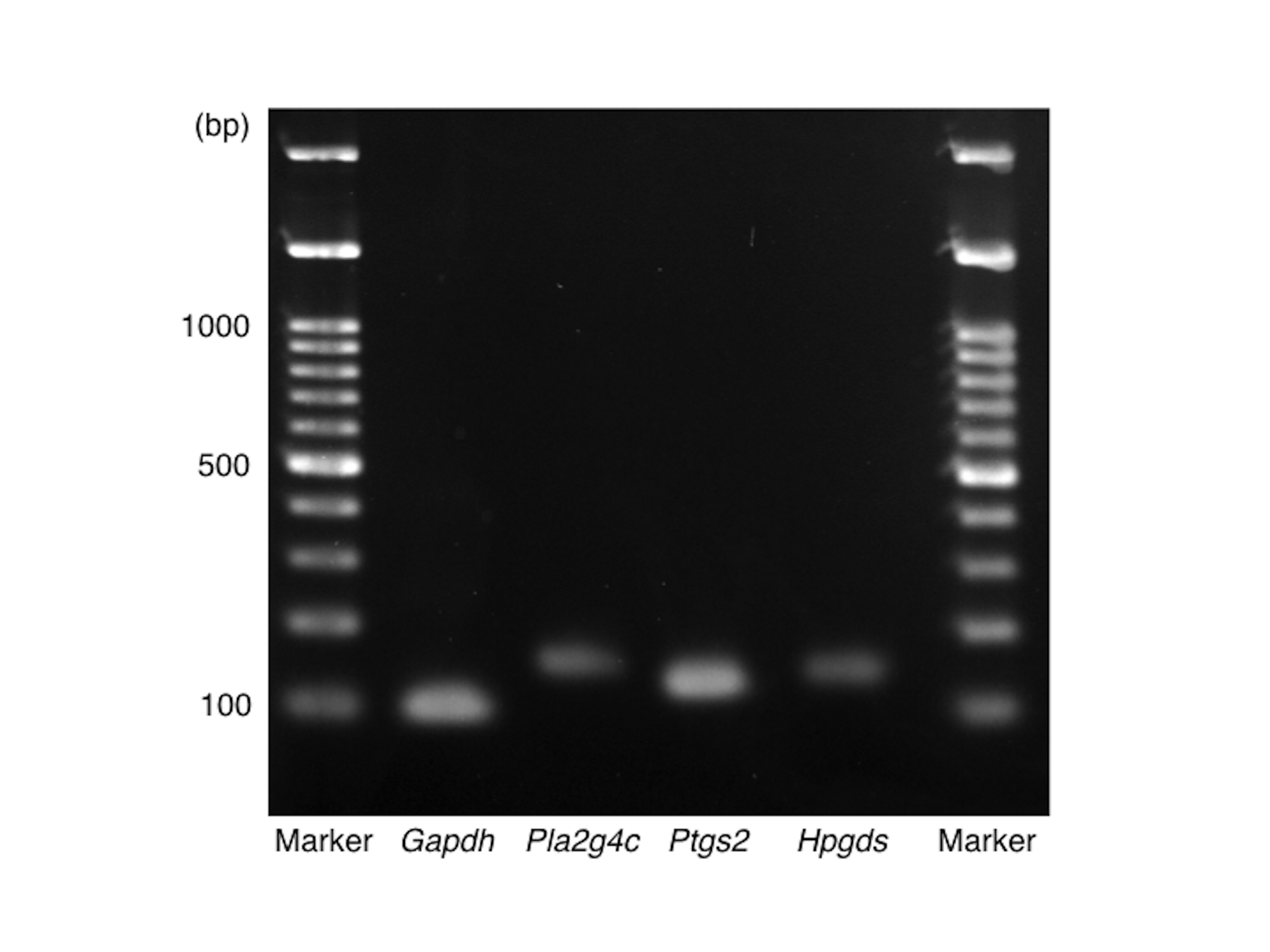

Supplement: S2 Fig — cDNA samples of the BSMs were amplified using specific primer sets for mouse Gapdh (forward primer: 5’-CCTCGTCCCGTAGACAAAATG-3’, reverse primer: 5’-TCTCCACTTTGCCACTGCAA-3’), Pla2g4c (forward primer: 5’-GGACCGTTGCGTTTTTGTGA-3’, reverse primer: 5’-GCAAAACCAGCATCCACCAG-3’), Ptgs2 (forward primer: 5’-CCGTGGGGAATGTATGAGCA-3’, reverse primer: 5’-GGGTGGGCTTCAGCAGTAAT-3’) and Hpgds (forward primer: 5’-TTCCCATGGGCAGAGAAAGA-3’, reverse primer: 5’-GCCCAGGTTACATAATTGCCT-3’), and detected by 2% agarose gel electrophoresis. Marker: M.W. markers (100 bp ladder). (TIFF) [file pone.0202623.s002.tiff]
